# Traumatic Hernia

**Published:** 1891-07

**Authors:** J. Wallace Marsh


### TRAUMATIC HERNIA.

---

BY J. WALLACE MARSH, D. V. S.

---

A ten days' old colt was injured by being hooked by a cow; the horn penetrated the abdominal cavity on the right side about three inches from the median line. The intestines escaped and reached nearly to the ground. The intestines were covered with sawdust from the litter of the paddock, they were greatly congested and swollen; the mesentery was torn and showed a hole an inch in diamater. I saw the colt about an hour after the injury and gave the following treatment It was laid on a linen sheet, the intestines were carefully washed under a carbolized spray ; the opening was enlarged and the intestines were slowly returned. The internal coverings were closed by buried cat-gut sutures, and the external by quilled suture. The wound was then covered by antiseptic cotton and the abdomen was wrapped with a wide linen bandage, which was well secured. The colt was kept on its side until the next morning, when it was assisted to its feet, but after that required no assistance. The dressing was not disturbed for forty-eight hours, when the wound was found closed. The quilled sutures were removed after four days. No swelling was present at any time; the wound was kept covered with antiseptic cotton. At the end of fourteen days the wound had completely healed and the colt was allowed its liberty. The colt and mare were then turned to pasture.

This proves to me that where the cat-gut suture and the thorough use of antiseptic dressings are employed, the least interference with the wound the better, especially in injuries of this nature.

